# Association of table salt use with sleep patterns and depressive symptoms: population-based analysis with external clinical replication

**DOI:** 10.3389/fnut.2026.1773531

**Published:** 2026-03-25

**Authors:** Qilong Wang, Siheng Ma, Xin Qi, Min Cai, Kairuo Ma, Lei Zhang, Sha Liu, Yuxia Jia, Tianci Liang, Weicong Liang, Dongrong Zhao, Jiwei Chen

**Affiliations:** 1 Department of Psychiatry, Gansu Provincial People’s Hospital, Lanzhou, Gansu, China; 2 The First Clinical College, Gansu University of Chinese Medicine, Lanzhou, Gansu, China; 3 Department of Psychiatry, Xijing Hospital, The Fourth Military Medical University, Xi’an, Shaanxi, China; 4 University of Illinois Urbana-Champaign, Urbana and Champaign, IL, United States; 5 Xi’an Medical University, Xi’an, Shaanxi, China

**Keywords:** depressive symptoms, dual-cohort study, mediation analysis, NHANES, sleep pattern, table salt use

## Abstract

**Background:**

Dietary habits are modifiable factors related to sleep health, yet the association between discretionary table-salt use and sleep patterns—and the potential role of depressive symptoms—remains incompletely understood. We investigated the association between table-salt use and sleep patterns and assessed whether depressive symptoms may statistically account for part of this association.

**Methods:**

This dual-cohort study integrated a nationally representative analysis of 8,440 adults from the National Health and Nutrition Examination Survey (NHANES, 2005–2010) with an independent external clinical replication cohort of 488 patients from Gansu Provincial People’s Hospital. The exposure was the frequency of adding salt at the table, and the outcome was an unhealthy sleep pattern (a composite score derived from sleep duration, trouble sleeping, and diagnosed sleep disorder). Mediation analyses were performed to quantify the indirect association component via depressive symptoms (PHQ-9).

**Results:**

In NHANES, frequent table salt use was independently associated with higher odds of unhealthy sleep patterns compared with rare/occasional use (OR = 1.27, 95% CI: 1.11–1.46; *p* < 0.001). In the external clinical replication cohort, the association showed a consistent direction but did not reach conventional statistical significance after full adjustment (OR = 1.62, 95% CI: 1.00–2.63; *p* = 0.052). Mediation analyses suggested that depressive symptoms statistically accounted for part of the salt–sleep association, representing 43.1% of the total association in NHANES and 55.8% in the external clinical replication cohort, which may reflect stronger symptom overlap in high-risk clinical settings. These findings should be interpreted as exploratory rather than causal. Subgroup analyses consistently identified young adults (aged 20–45 years) as a particularly vulnerable population.

**Conclusion:**

More frequent discretionary table salt use was associated with unhealthy sleep patterns in a nationally representative sample, with directionally consistent findings in an external clinical replication cohort. Depressive symptoms statistically accounted for a substantial proportion of the observed association. Given the cross-sectional design, temporality cannot be established, and mediation results should be interpreted as a statistical decomposition of associations rather than causal mediation. Prospective studies with repeated measurements and intervention trials are needed to test temporal ordering and causal mechanisms.

## Graphical abstract

**Figure:**
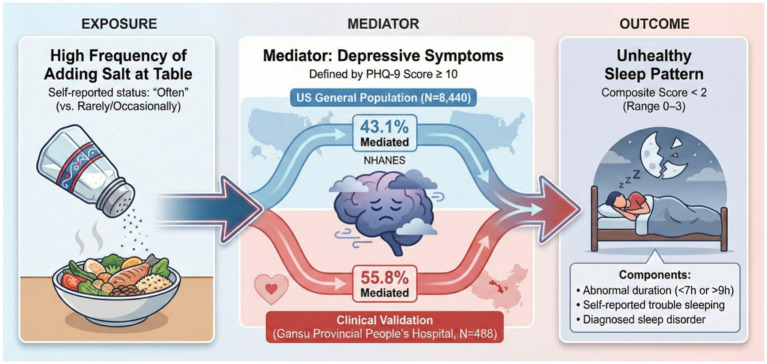

## 1 Introduction

Sleep, a fundamental component of human life, is intricately associated with physical and mental well-being, accounting for approximately one-third of an individual’s lifespan (1). It is estimated that approximately 1 billion people worldwide suffer from sleep disorders, and the increasing prevalence of this condition is having a seriously detrimental effect on the quality of life for many individuals (2). Despite the National Sleep Foundation’s recommendation that adults aged 18 years and over should obtain a minimum of 7 h of sleep per night (3), a survey revealed that 30% of Americans fail to meet this standard (4). Numerous studies have demonstrated that sleep disorders are linked to a range of conditions, including depression (5), cognitive impairment (6), coronary artery disease and stroke (7). Sleep problems not only significantly impact quality of life but also adversely affect physical and mental health. Therefore, there is an urgent need to prioritize improving sleep to reduce the associated health risks.

Traditionally, the management of sleep disorders has primarily focused on pharmacological interventions, including benzodiazepines and non-benzodiazepine agonists (8). However, the effectiveness of these interventions is often compromised by side effects, the potential for dependence, and, in some cases, their limited efficacy in addressing the underlying causes of sleep disorders (9). Among non-pharmacological interventions, lifestyle modifications, particularly dietary changes, have been acknowledged as effective treatment options (10). Studies have demonstrated a significant association between sleep and diet; therefore, improving nutritional habits may be an effective intervention strategy (11–14). Discretionary salt-use habits warrant attention. Previous studies have primarily examined the effects of a high-salt diet on the cardiovascular, immune, and gut microbiota systems (15–18); however, the impact of a high-salt diet on physical and mental health should not be overlooked. Evidence suggests that higher dietary sodium exposure is associated with adverse neurocognitive correlates and may be linked to poorer sleep quality (19–21).

Although prior studies have reported associations between sodium-related dietary exposure and sleep outcomes, the mechanisms underlying these relationships remain incompletely understood. A plausible hypothesis is that a high-salt diet may influence mood and sleep quality by altering dopamine synthesis, enhancing dopamine neurons’ activity, and affecting dopamine receptors (22–24). Furthermore, there is a significant relationship between depression and poor sleep quality, with depression not only potentially causing sleep disorders but also being triggered by them (25). This bidirectional relationship may involve disruptions in circadian rhythms, increased oxidative stress, and activation of the hypothalamic–pituitary–adrenal (HPA) axis, all of which play important roles in sleep regulation (26). Based on this pattern of associations, we hypothesize that depressive symptoms may partly account for the association between table salt use and sleep patterns. While complex interactions among diet, depression, and sleep are well-documented, the specific mediating role of depression in the relationship between salt use habits and sleep patterns remains underexplored. Most research has emphasized the physiological correlates of sodium intake, with less attention to potential psychological factors that may be linked to these associations. This gap hinders the development of effective dietary interventions for mental and sleep health. To address this, we investigated the mediating role of depressive symptoms in the association between salt use and sleep patterns. We hypothesized that higher discretionary salt use is associated with unhealthy sleep patterns, with depressive symptoms accounting for part of this association. NHANES provides population representativeness, and our findings were replicated in an independent external clinical replication cohort from Gansu Provincial People’s Hospital, supporting reproducibility in a real-world clinical context.

## 2 Methods

### 2.1 Study population

To enhance robustness and assess reproducibility across settings, this study employed a two-stage design comprising a primary analysis of a nationally representative U.S. population from the National Health and Nutrition Examination Survey (NHANES) and an independent external clinical replication cohort from China (Gansu Provincial People’s Hospital). NHANES is a continuous cross-sectional study conducted by the National Center for Health Statistics (NCHS) using a complex, multistage, stratified probability sampling design. We merged data from three biennial cycles (2005–2010) to maximize the sample size of participants with complete data on table salt use (frequency of adding salt at the table), multidimensional sleep health metrics (sleep duration, trouble sleeping, and sleep disorder), and PHQ-9 depressive symptom evaluations. The 2005–2010 cycles were selected because they were the most recent cycles in which comparable measures of table salt use, PHQ-9, and all sleep components required for this study were concurrently available. In NHANES, these measures were collected within the same survey cycle for each participant. Specifically, sleep-related items were obtained during the in-home interview, whereas PHQ-9 depressive symptoms and dietary measures (including table salt use) were assessed during the Mobile Examination Center (MEC) visit (dietary component), thereby capturing these variables within a short, cycle-specific assessment window. The inclusion criteria for the NHANES cohort were age ≥20 years and complete documentation of the aforementioned variables. Participants were excluded if they were pregnant, had a history of malignancies or end-stage renal disease, or had missing data on key covariates. All NHANES protocols were approved by the NCHS Research Ethics Review Board, and informed consent was obtained from all participants.

**Figure 1:**
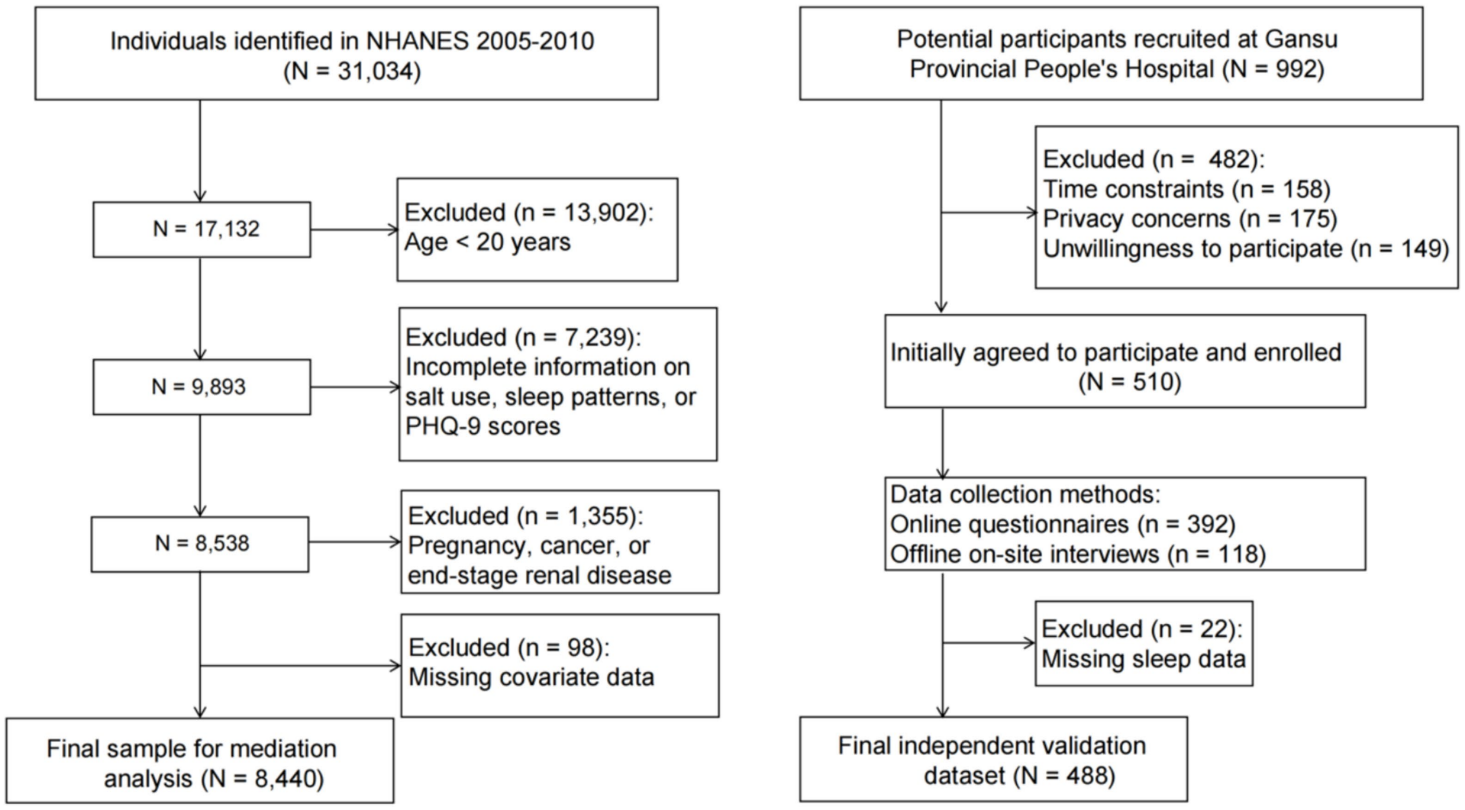
Flowchart of participants in this study.

For the external clinical replication cohort, we recruited an independent clinical cohort from the Department of Psychiatry at Gansu Provincial People’s Hospital between June 1, 2025, and November 30, 2025. Given the clinical setting, a convenience sampling method was utilized. Potential participants (*n* = 992) aged ≥20 years presenting with mood-related concerns were identified through outpatient and inpatient registration systems. Following a detailed explanation of the study, 482 individuals declined to participate due to privacy concerns (*n* = 175), time conflicts (*n* = 158), or lack of willingness (*n* = 149). Ultimately, 510 participants provided informed consent and were enrolled. To maximize response rates, data were collected using a hybrid approach combining online questionnaires and offline on-site interviews. The final replication analysis included 488 participants who met the strict inclusion criteria: age ≥20 years, and complete data on psychological screening, sleep metrics, salt usage, and all covariates (22 participants were excluded due to incomplete data). This replication study was approved by the Ethics Committee of Gansu Provincial People’s Hospital (Approval No. 2025-439), and all participants consented to the anonymous use of their data. The participant selection process is detailed in Figure 1.

### 2.2 Assessment of depression (mediator)

Each PHQ-9 item is intended to represent a specific depressive symptom, with scores ranging from 0 (not at all) to 3 (almost every day) (27). The total PHQ-9 score ranges from 0 to 27. The prevailing consensus among preceding studies indicates that the optimal threshold for major depression is a score of 10 on the PHQ-9, with a sensitivity and specificity of 88% (27). Thus, in the present study, a PHQ-9 score of ≥10 was used to indicate the presence of current depressive symptoms. To ensure diagnostic rigor in the Gansu Provincial People’s Hospital clinical cohort, senior psychiatrists further validated these symptoms through structured clinical interviews according to the Diagnostic and Statistical Manual of Mental Disorders, Fifth Edition (DSM-5) criteria (28). This two-step approach—combining standardized scale screening with expert clinical confirmation—enhances the rigor of findings in the external clinical replication cohort.

### 2.3 Assessment of frequency of adding salt at the table (exposure)

Data on the frequency of adding extra salt to food at the table was gathered from participants using a baseline questionnaire that asked, “How often do you add extra salt to food at the table? (omit cooking salt).” Participants selected one of three options: (1) rarely/occasionally, (2) often, or (3) decline to answer. Those who selected “decline to answer” were classified as part of the missing value group (29). While 24-h urinary sodium is the gold standard for total intake, this frequency-based metric was selected as a practical proxy for long-term discretionary salt-use behavior and taste preferences. It offers greater clinical operability for large-scale screening and direct behavioral interventions than biochemical measures. Nevertheless, this measure does not quantify total dietary sodium intake (including salt added during cooking or sodium from processed foods) and may be influenced by eating practices and taste preference. Therefore, it should be interpreted as an indicator of discretionary salt-use behavior (salt preference) rather than a biomarker of total sodium exposure, particularly when comparing NHANES with a Chinese clinical cohort. Data collection for the external replication cohort followed an identical procedure.

### 2.4 Assessment of sleep pattern (outcome)

Sleep assessment included three sleep-related components—sleep duration, self-reported trouble sleeping, and physician-diagnosed sleep disorder—and a composite sleep pattern derived from these components. Sleep duration was assessed using an NHANES questionnaire item asking, “How much sleep do you get (hours)?”, with responses ranging from 1 to 12 h. Trouble sleeping was assessed using the item “Ever told doctor had trouble sleeping?”, and sleep disorder was assessed using “Ever told by doctor have sleep disorder?”, with response options of yes/no/refused/do not know (refused/do not know were treated as missing). Given that sleep health is conceptualized as a multidimensional construct, we constructed a composite sleep pattern score by summing three binary indicators: sleep duration (7–9 h = 1; <7 or >9 h = 0), trouble sleeping (no = 1; yes = 0), and sleep disorder (no = 1; yes = 0). The total score ranged from 0 to 3, with ≥2 indicating a healthy sleep pattern and <2 indicating an unhealthy sleep pattern (11). Data collection for the external replication cohort followed the same procedure, using the same classification criteria.

### 2.5 Assessment of covariates

The following covariates were included in this study: gender, age, race, marital status, education, household income to poverty ratio (PIR), body mass index (BMI, kg/m^2^), smoking status, alcohol consumption status, and personal history of hypertension, hyperlipidemia, diabetes, and cardiovascular disease. These covariates were selected *a priori* by referring to previous studies with similar designs and comparable exposure/outcome measures, adopting their commonly adjusted confounders (30, 31). In the NHANES cohort, smoking status was determined through home interviews, categorizing participants as never smokers or smokers. Alcohol drinking status was defined as the consumption of an average of more than 5 alcoholic beverages per day in the 12 months preceding the interview. BMI was classified into four categories: underweight (<18.5 kg/m^2^), normal weight (18.5–24.9 kg/m^2^), overweight (25–29.9 kg/m^2^), and obese (≥30 kg/m^2^). Chronic conditions (hypertension, hyperlipidemia, diabetes mellitus, and cardiovascular disease) were identified based on self-reported verbal confirmation of a diagnosis by a physician or other health professional. For the external replication cohort, covariates were assessed using identical definitions and categorization standards to ensure comparability, except for race, which was excluded due to the homogeneity of the study population.

### 2.6 Statistical analysis

Given the complex, multistage, stratified probability sampling design of NHANES, all primary analyses accounted for sample weights, stratification, and primary sampling units (PSUs) to ensure national representativeness. Continuous variables were expressed as weighted means ± standard deviations (SD), and categorical variables as weighted percentages (SD). Differences between groups (healthy vs. unhealthy sleep patterns) were evaluated using weighted t-tests for continuous variables and weighted chi-square tests for categorical variables. We employed multivariable logistic regression models to examine the associations among table salt use, depressive symptoms, and sleep patterns. The mediating role of depressive symptoms in the salt–sleep relationship was analyzed using the R mediation package with quasi-Bayesian Monte Carlo approximation. Mediation analysis relies on correct model specification and the assumption of sequential ignorability (i.e., no unmeasured confounding of the exposure–mediator, exposure–outcome, and mediator–outcome relationships conditional on included covariates), as well as consistency and positivity. We addressed these requirements by specifying separate mediator and outcome models and adjusting for *a priori* confounders commonly used in similar studies. Sensitivity analyses included stratified and interaction analyses across key subgroups, component-specific analyses of the composite sleep outcome (sleep duration, trouble sleeping, and sleep disorders), a measurement-overlap analysis using a modified PHQ-8 score (PHQ-9 excluding the sleep item) as the mediator, and a disease-exclusion analysis repeating the mediation models after excluding participants with baseline CVD or diabetes mellitus. Importantly, because both NHANES and the external clinical replication cohort are cross-sectional, temporality among exposure (table salt use), mediator (depressive symptoms), and outcomes (sleep metrics) cannot be established. Therefore, the mediation estimates represent statistical mediation of observed associations under the specified models and should not be interpreted as causal mediation effects.

For the external clinical replication cohort, descriptive statistics were performed using standard methods without sampling weights. Continuous variables were presented as means (SE) or medians (interquartile range, IQR) based on distribution, and categorical variables as frequencies (percentages). Disparities between sleep pattern groups were assessed using Student’s *t*-tests or chi-square tests. To verify the consistency of the primary findings, multivariate logistic regression and subgroup analyses were replicated in this cohort using identical model specifications. We further performed post-hoc power analyses to quantify the capacity to detect true effects in the external clinical replication cohort; with the achieved sample size (*n* = 488) and a significance threshold of *α* = 0.05, the statistical power for the primary outcomes exceeded 90%, surpassing the recommended 80% threshold. Furthermore, to assess the robustness of the core outcome (unhealthy sleep patterns) against potential unmeasured confounding, the *E*-value was calculated.

All statistical analyses were performed using R version 4.4.2 (The R Foundation) and EmpowerStats (X&Y Solutions, Inc., Boston, MA), with a two-tailed *p*-value <0.05 considered statistically significant.

## 3 Results

### 3.1 Description of study participants

Table 1 summarizes the baseline characteristics of the study participants. Among the 8,440 NHANES participants included in the analysis, 1,311 (15.5%) were classified as having unhealthy sleep patterns. Demographically, the unhealthy sleep group was significantly older (mean age: 50.48 vs. 45.98 years) and comprised a higher proportion of females and divorced or widowed individuals compared to the healthy sleep group (all *p* < 0.05). Regarding health behaviors and comorbidities, the unhealthy sleep group carried a markedly heavier disease burden: the prevalence of obesity, smoking, and chronic conditions—including hypertension, diabetes, and cardiovascular disease—was significantly higher than in the healthy group (all *p* < 0.001). Notably, alcohol consumption was significantly lower in this group compared to the healthy group (*p* = 0.011). Crucially, key study variables showed significant intergroup differences: the unhealthy sleep group reported a higher rate of “frequently” adding extra salt (27.46% vs. 24.21%, *p* = 0.012). Additionally, the prevalence of depressive symptoms reached 47.52%, substantially higher than the 16.94% observed in the healthy group (*p* < 0.001). The only non-significant difference between the groups in the NHANES cohort was the PIR (*p* = 0.216).

**Table 1:** Characteristics of participants by sleep pattern in the baseline.

Variables	NHANES				Gansu Provincial People’s Hospital			
	Total (*n* = 8,440)	Healthy sleep (*n* = 7,129)	Unhealthy sleep (*n* = 1,311)	*p*	Total (*n* = 488)	Healthy sleep (*n* = 357)	Unhealthy sleep (*n* = 131)	*p*
Age, mean ± SD	46.68 ± 17.04	45.98 ± 17.23	50.48 ± 15.47	<0.001	35.05 ± 8.29	33.95 ± 7.88	38.04 ± 8.67	<0.001
Gender, *n* (%)				<0.001				<0.001
Male	4,420 (52.37)	3,819 (53.57)	601 (45.84)		224 (45.90)	180 (50.42)	44 (33.59)	
Female	4,020 (47.63)	3,310 (46.43)	710 (54.16)		264 (54.10)	177 (49.58)	87 (66.41)	
Race, *n* (%)				<0.001	/	/	/	/
Mexican American	1,733 (20.53)	1,556 (21.83)	177 (13.50)					
Other Hispanic	660 (7.82)	558 (7.83)	102 (7.78)					
Non-Hispanic White	4,088 (48.44)	3,385 (47.48)	703 (53.62)					
Non-Hispanic Black	1,623 (19.23)	1,340 (18.80)	283 (21.59)					
Other race	336 (3.98)	290 (4.07)	46 (3.51)					
Education, *n* (%)				0.02				0.374
Less than high school	2,344 (27.77)	2,009 (28.18)	335 (25.55)		24 (4.92)	18 (5.04)	6 (4.58)	
High school or equivalent	2,117 (25.08)	1,751 (24.56)	366 (27.92)		68 (13.93)	45 (12.61)	23 (17.56)	
College or above	3,979 (47.14)	3,369 (47.26)	610 (46.53)		396 (81.15)	294 (82.35)	102 (77.86)	
Marital status, *n* (%)				<0.001				0.029
Married or living with a partner	5,162 (61.16)	4,431 (62.15)	731 (55.76)		318 (65.16)	225 (63.03)	93 (70.99)	
Never married	1,573 (18.64)	1,363 (19.12)	210 (16.02)		148 (30.33)	119 (33.33)	29 (22.14)	
Widowed/Divorced/Separated	1,705 (20.20)	1,335 (18.73)	370 (28.22)		22 (4.51)	13 (3.64)	9 (6.87)	
PIR, *n* (%)				0.216				0.905
<130%	2,345 (27.78)	1,955 (27.42)	390 (29.75)		254 (52.05)	185 (51.82)	69 (52.67)	
130–349%	3,527 (41.79)	2,990 (41.94)	537 (40.96)		200 (40.98)	148 (41.46)	52 (39.69)	
≥350%	2,568 (30.43)	2,184 (30.64)	384 (29.29)		34 (6.97)	24 (6.72)	10 (7.63)	
BMI, *n* (%)				<0.001				0.073
Underweight (<18.5)	130 (1.54)	114 (1.60)	16 (1.22)		26 (5.33)	22 (6.16)	4 (3.05)	
Normal weight (18.5 to 24.9)	2,469 (29.25)	2,190 (30.72)	279 (21.28)		280 (57.38)	212 (59.38)	68 (51.91)	
Overweight (25 to 29.9)	2,877 (34.09)	2,488 (34.90)	389 (29.67)		136 (27.87)	95 (26.61)	41 (31.30)	
Obese (≥30)	2,964 (35.12)	2,337 (32.78)	627 (47.83)		46 (9.43)	28 (7.84)	18 (13.74)	
Smoking status, *n* (%)				<0.001				0.027
No	4,296 (50.90)	3,739 (52.45)	557 (42.49)		416 (85.25)	312 (87.39)	104 (79.39)	
Yes	4,144 (49.10)	3,390 (47.55)	754 (57.51)		72 (14.75)	45 (12.61)	27 (20.61)	
Alcohol drinking status, *n* (%)				0.011				<0.001
No	7,273 (86.17)	6,114 (85.76)	1,159 (88.41)		350 (71.72)	273 (76.47)	77 (58.78)	
Yes	1,167 (13.83)	1,015 (14.24)	152 (11.59)		138 (28.28)	84 (23.53)	54 (41.22)	
Depression, *n* (%)				<0.001				<0.001
No	6,609 (78.31)	5,921 (83.06)	688 (52.48)		387 (79.30)	314 (87.96)	73 (55.73)	
Yes	1,831 (21.69)	1,208 (16.94)	623 (47.52)		101 (20.70)	43 (12.04)	58 (44.27)	
CVD, *n* (%)				<0.001				0.001
No	7,774 (92.11)	6,666 (93.51)	1,108 (84.52)		460 (94.26)	344 (96.36)	116 (88.55)	
Yes	666 (7.89)	463 (6.49)	203 (15.48)		28 (5.74)	13 (3.64)	15 (11.45)	
Diabetes, *n* (%)				<0.001				<0.001
No	7,557 (89.54)	6,505 (91.25)	1,052 (80.24)		470 (96.31)	352 (98.60)	118 (90.08)	
Yes	883 (10.46)	624 (8.75)	259 (19.76)		18 (3.69)	5 (1.40)	13 (9.92)	
Hypertension, *n* (%)				<0.001				0.027
No	6,034 (71.49)	5,338 (74.88)	696 (53.09)		450 (92.21)	335 (93.84)	115 (87.79)	
Yes	2,406 (28.51)	1,791 (25.12)	615 (46.91)		38 (7.79)	22 (6.16)	16 (12.21)	
High cholesterol, *n* (%)				<0.001				0.001
No	3,170 (37.56)	2,668 (37.42)	502 (38.29)		402 (82.38)	306 (85.71)	96 (73.28)	
Yes	2,325 (27.55)	1,784 (25.02)	541 (41.27)		86 (17.62)	51 (14.29)	35 (26.72)	
Missing	2,945 (34.89)	2,677 (37.55)	268 (20.44)					
Table salt use, *n* (%)				0.012				0.011
Rarely/Occasionally	6,354 (75.28)	5,403 (75.79)	951 (72.54)		330 (67.62)	253 (70.87)	77 (58.78)	
Often	2,086 (24.72)	1,726 (24.21)	360 (27.46)		158 (32.38)	104 (29.13)	54 (41.22)	

Mean ± SE for continuous variables: *p*-value was calculated by the weighted linear regression model. *n* (%) for categorical variables: *p*-value was calculated by the weighted chi-square test.

BMI, body mass index; PIR, poverty-income ratio; CVD, cardiovascular disease.

The replication cohort from Gansu Provincial People’s Hospital (*n* = 488) confirmed these primary trends. Similarly, the unhealthy sleep group was characterized by older age, a female predominance, higher smoking rates, and a greater burden of chronic diseases. Most importantly, this cohort validated the core associations: the unhealthy sleep group exhibited significantly higher rates of “frequent” salt addition (41.22% vs. 29.13%, *p* = 0.011) and a higher prevalence of depressive symptoms (44.27% vs. 12.04%, *p* < 0.001).

However, some disparities were noted between the cohorts. In the replication cohort, alcohol consumption was significantly higher in the unhealthy sleep group than in the healthy group (*p* < 0.001), presenting a trend opposite to that observed in NHANES. Furthermore, no significant differences were found in the replication cohort regarding educational attainment (*p* = 0.374) or BMI (*p* = 0.073).

### 3.2 Association between table salt use and sleep pattern

Table 2 presents the results of the multivariable logistic regression analysis examining the association between the frequency of table salt use and unhealthy sleep patterns. In the NHANES cohort, a consistent positive association was observed across all models. In the unadjusted model (Model 1), participants who reported adding salt “often” had a significantly higher risk of unhealthy sleep patterns compared to those who did so “rarely or occasionally” (OR = 1.18, 95% CI: 1.04, 1.35, *p* = 0.012). After adjusting for demographic factors, including age, gender, and marital status (Model 2), the association remained significant (OR = 1.22, 95% CI: 1.06–1.39, *p* = 0.004). In the fully adjusted model (Model 3), the association strengthened slightly, with an OR of 1.27 (95% CI: 1.11, 1.46, *p* < 0.001). These findings were corroborated by the independent replication cohort from Gansu Provincial People’s Hospital. The unadjusted model demonstrated a substantial association (OR = 1.71, 95% CI: 1.13, 2.59, *p* = 0.012). Adjusting for demographic variables (Model 2) further increased the magnitude of the association (OR = 2.13, 95% CI: 1.37, 3.32, *p* < 0.001). Crucially, in the fully adjusted model (Model 3), the association remained directionally consistent but did not reach conventional statistical significance (OR = 1.62, 95% CI: 1.00–2.63; *p* = 0.052).

**Table 2:** Logistic regression between table salt use and unhealthy sleep patterns.

	NHANES			Gansu Provincial People’s Hospital		
Table-salt use	Model 1	Model 2	Model 3	Model 1	Model 2	Model 3
	OR (95% CI) *p*-value	OR (95% CI) *p*-value	OR (95% CI) *p*-value	OR (95% CI) *p*-value	OR (95% CI) *p*-value	OR (95% CI) *p*-value
Rarely/Occasionally	Reference	Reference	Reference	Reference	Reference	Reference
Often	1.18 (1.04, 1.35) 0.012	1.22 (1.06, 1.39) 0.004	1.27 (1.11, 1.46) < 0.001	1.71 (1.13, 2.59) 0.012	2.13 (1.37, 3.32) < 0.001	1.62 (1.00, 2.63) 0.052

Model 1: unadjusted.

Model 2: Model 1 + age, gender, marital status.

Model 3: Model 2 + smoking status, alcohol drinking status, CVD, diabetes, hypertension, and high cholesterol.

CVD, cardiovascular disease.

### 3.3 Association between table salt use and depressive symptoms

Table 3 presents the results of the multivariable logistic regression analysis assessing the relationship between the frequency of table salt use and depressive symptoms. In the NHANES cohort, Table 3 indicates that frequent table salt use exhibits a significant positive association with depressive symptoms. In the unadjusted model (Model 1), the likelihood of depressive symptoms among those who frequently added salt increased by 37% (OR = 1.37, 95% CI: 1.22–1.53, *p* < 0.001). This association remained robust in Model 2 after adjusting for age, gender, and marital status (OR = 1.35, 95% CI: 1.20–1.52, *p* < 0.001). In the fully adjusted Model 3, the OR remained stable at 1.38 (95% CI: 1.23–1.56, *p* < 0.001), confirming the independence of this association. In the replication cohort at Gansu Provincial People’s Hospital, the association was even more pronounced. The odds of depressive symptoms in the frequent salt-adding group were elevated by 2.55-fold in the unadjusted model (Model 1) (95% CI: 1.63–3.98, *p* < 0.001). In Model 2, adjusted for demographic variables, this estimate further increased to 2.91-fold (95% CI: 1.83–4.62, *p* < 0.001). Even in the fully adjusted model (Model 3), which comprehensively adjusted for all potential confounders, the risk remained significantly higher than that of the reference group (OR = 2.34, 95% CI: 1.43–3.85, *p* < 0.001).

**Table 3:** Logistic regression between table salt use and depressive symptoms.

	NHANES			Gansu Provincial People’s Hospital		
Table-salt use	Model 1	Model 2	Model 3	Model 1	Model 2	Model 3
	OR (95% CI) *p*-value	OR (95% CI) *p*-value	OR (95% CI) *p*-value	OR (95% CI) *p*-value	OR (95% CI) *p*-value	OR (95% CI) *p*-value
Rarely/Occasionally	Reference	Reference	Reference	Reference	Reference	Reference
Often	1.37 (1.22, 1.53) < 0.001	1.35 (1.20, 1.52) < 0.001	1.38 (1.23, 1.56) < 0.001	2.55 (1.63, 3.98) < 0.001	2.91 (1.83, 4.62) < 0.001	2.34 (1.43, 3.85) < 0.001

Model 1: unadjusted.

Model 2: Model 1 + age, gender, marital status.

Model 3: Model 2 + smoking status, alcohol drinking status, CVD, diabetes, hypertension, and high cholesterol.

### 3.4 The mediating role of depressive symptoms in table salt use and sleep

**Figure 2:**
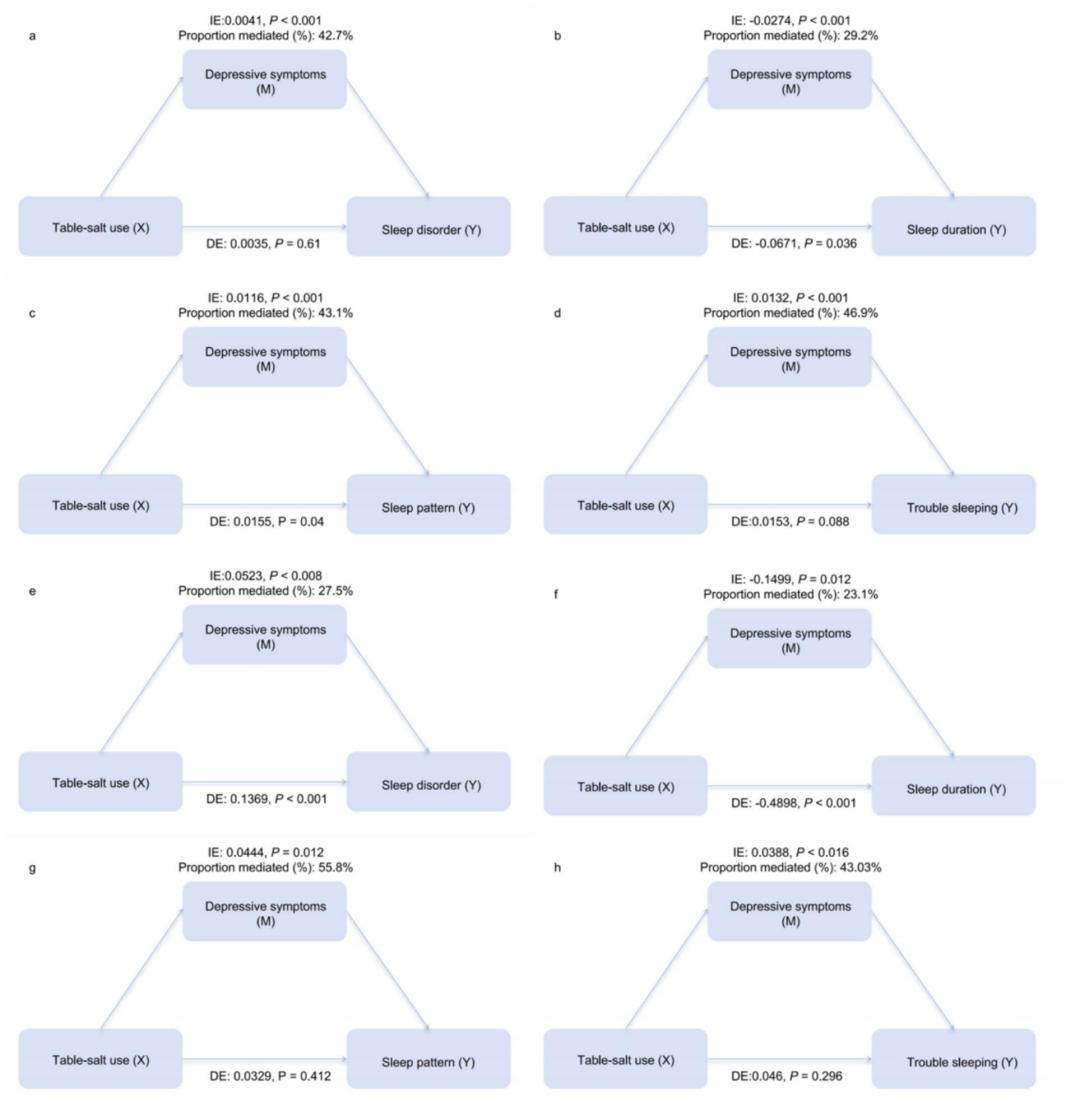
Mediation analysis of depressive symptoms in the association between the frequency of table salt use and sleep outcomes. The upper panels display results from the NHANES cohort: **(a)** sleep disorder, **(b)** sleep duration, **(c)** sleep pattern, and **(d)** trouble sleeping. The lower panels display results from the Gansu Provincial People’s Hospital replication cohort: **(e)** sleep disorder, **(f)** sleep duration, **(g)** sleep pattern, and **(h)** trouble sleeping. Numbers on the paths represent the effect estimates. IE: indirect effect (pathway via depressive symptoms); DE: direct effect (pathway independent of depressive symptoms). All mediation models were adjusted for age, gender, marital status, smoking status, alcohol drinking status, CVD, diabetes, hypertension, and high cholesterol.

Figure 2 illustrates the results of the path analysis examining depressive symptoms as a mediator. In the NHANES cohort, depressive symptoms played a significant mediating role. For the composite sleep pattern and sleep duration, both the indirect and direct components were statistically significant (all *p* < 0.05), consistent with partial mediation, with proportions mediated (PM) of 43.1 and 29.2%, respectively. For sleep disorder and trouble sleeping, the indirect components were statistically significant (all *p* < 0.001), whereas the direct effects did not reach statistical significance (*p* = 0.61 and *p* = 0.088, respectively), a pattern consistent with an indirect-only association via depressive symptoms (PM = 42.7 and 46.9%).

The replication cohort at Gansu Provincial People’s Hospital further corroborated these mechanisms, though with distinct variations in mediation patterns. For the primary outcome, sleep pattern, depressive symptoms exhibited a significant full mediation effect in this cohort (PM = 55.8%): the indirect effect was significant (*p* = 0.012), whereas the direct effect was not (*p* = 0.412). Trouble sleeping also presented a full mediation pattern (PM = 43.03%; direct effect *p* = 0.296). In contrast, sleep disorder (PM = 27.5%) and sleep duration (PM = 23.1%) retained partial mediation characteristics in this cohort, with both direct and indirect effects remaining statistically significant (all *p* < 0.05).

### 3.5 Sensitivity analysis

#### 3.5.1 Stratified and interaction analyses

**Figure 3:**
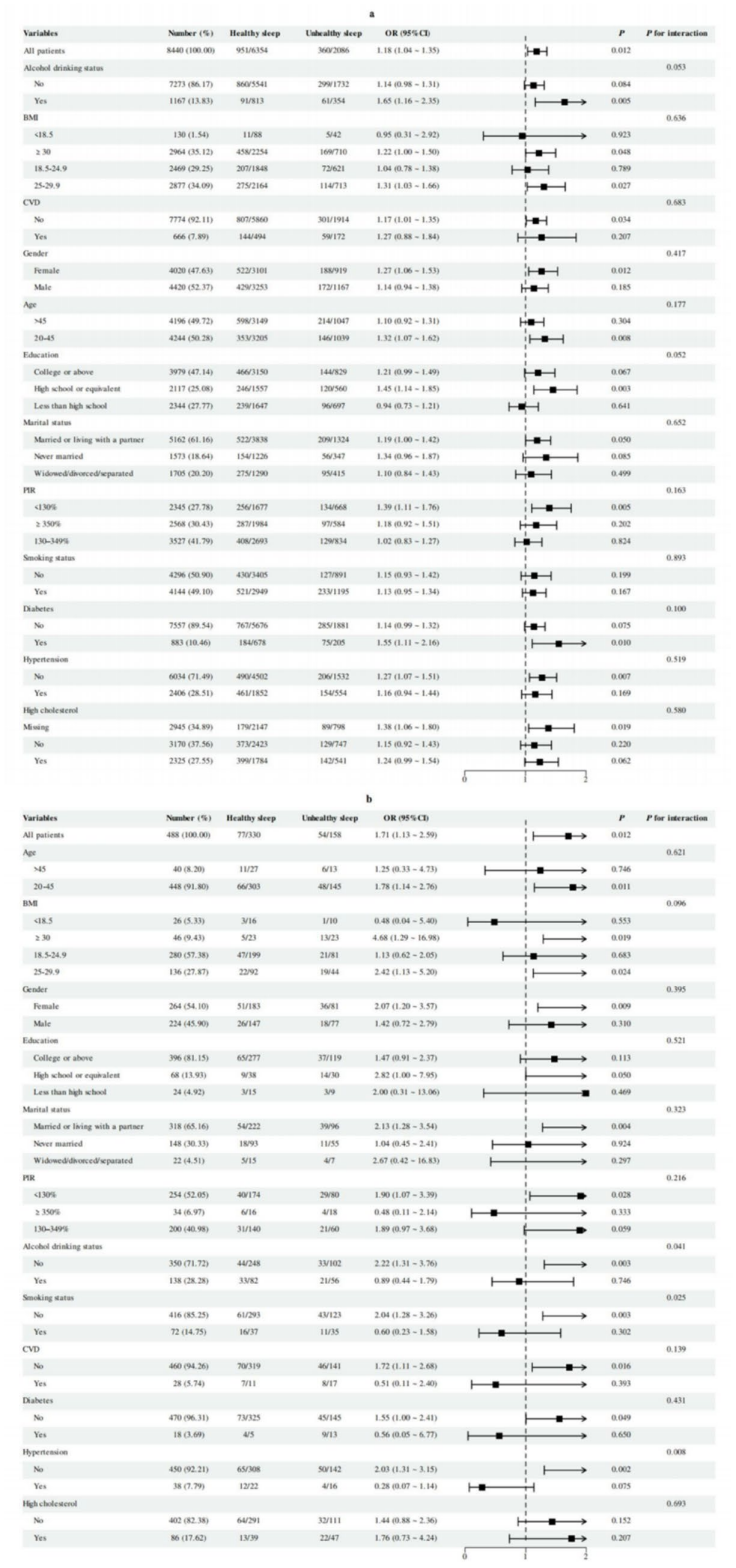
Subgroup analyses of the association between the frequency of table salt use and unhealthy sleep patterns: **(a)** results from the NHANES cohort and **(b)** results from the Gansu Provincial People’s Hospital replication cohort.

Within the NHANES cohort (Figure 3a), the association between frequent table salt addition and unhealthy sleep patterns was most evident among participants aged 20–45 years (OR = 1.32, 95% CI: 1.07–1.62; *p* = 0.008). Educational attainment and alcohol use showed suggestive effect modification (*p* for interaction = 0.052 and 0.053, respectively), with higher risks observed among high school graduates (OR = 1.45, 95% CI: 1.14–1.85; *p* = 0.003) and drinkers (OR = 1.65, 95% CI: 1.16–2.35; *p* = 0.005). Positive associations were also observed in women (OR = 1.27, 95% CI: 1.05–1.53; *p* = 0.012), participants with obesity (BMI ≥30; OR = 1.22, 95% CI: 1.00–1.48; *p* = 0.048), low income (PIR <130%; OR = 1.39, 95% CI: 1.10–1.76; *p* = 0.005), and diabetes (OR = 1.55, 95% CI: 1.11–2.16; *p* = 0.010).

In the Gansu Provincial People’s Hospital replication cohort (Figure 3b), the age-specific trend was reproduced, with significance only in the 20–45 age group (OR = 1.78, 95% CI: 1.14–2.76; *p* = 0.011). In contrast to NHANES, the association was stronger among non-drinkers (OR = 2.22, 95% CI: 1.31–3.76; *p* = 0.003; *p* for interaction = 0.041), and was also more evident among non-smokers (OR = 2.04, 95% CI: 1.28–3.26; *p* = 0.003; *p* for interaction = 0.025) and those without hypertension (OR = 2.03, 95% CI: 1.31–3.15; *p* = 0.002; *p* for interaction = 0.008). This discrepancy by drinking status may reflect contextual heterogeneity between cohorts and should be interpreted cautiously. Additional subgroup results are presented in Figure 3.

#### 3.5.2 Component-specific analyses of the composite sleep outcome

To assess robustness, we analyzed sleep duration, trouble sleeping, and sleep disorders as separate outcomes (Supplementary Tables 1–3). In NHANES, frequent table salt use was associated with a higher risk of trouble sleeping and shorter sleep duration. In contrast, the association with sleep disorder was not statistically significant after full adjustment. In the replication cohort, frequent salt use was associated with all three components, and the effect directions were broadly consistent with those in the primary analysis.

#### 3.5.3 Measurement-overlap sensitivity analysis (modified PHQ-8)

To minimize measurement overlap, we repeated the mediation analysis using a modified PHQ-8 score (PHQ-9, excluding item 3, the sleep item) as the mediator (Supplementary Figure 1). In NHANES, the indirect component persisted but was attenuated compared with PHQ-9. In the external clinical replication cohort, removing the sleep item altered the mediation pattern, underscoring the need for cautious interpretation of mediation estimates.

#### 3.5.4 Disease-exclusion sensitivity analysis

To mitigate potential confounding from CVD, diabetes, and other conditions that may interfere with mood, we excluded individuals with these diseases and repeated the mediation analysis. The results showed that the mediation patterns were largely consistent with the primary analysis, further validating the robustness of our findings.

## 4 Discussion

In this dual-cohort analysis, including a nationally representative sample of 8,440 U.S. adults (NHANES) and an independent clinical cohort of 488 participants from Gansu Provincial People’s Hospital, more frequent discretionary salt use (adding salt at the table) was associated with higher odds of unhealthy sleep patterns after adjustment for a broad set of covariates. Mediation analyses further indicated that depressive symptoms statistically accounted for a substantial proportion of this association in both cohorts (43.1% in NHANES and 55.8% in the clinical cohort), and subtype analyses showed broadly consistent patterns across sleep components. Although the components reflect distinct domains, the component-specific analyses showed consistent directions, suggesting that the composite measure captures an overall poor-sleep phenotype. Dichotomization may be associated with information loss, which should be considered when interpreting effect sizes. Because both datasets are cross-sectional, the temporal ordering and directionality among discretionary salt use, depressive symptoms, and sleep outcomes cannot be established; therefore, these findings should be interpreted as correlational and hypothesis-generating, and the mediation results should be viewed as statistical decompositions of associations rather than evidence of a causal pathway.

Adding salt to the table may reflect a behavioral preference for salty taste and related dietary tendencies (27), which could help generate hypotheses about behavioral and neurobiological mechanisms linking diet, mood regulation, and sleep. It should be noted that “table salting frequency” reflects taste preferences and discretionary salt use rather than directly quantifying total dietary sodium intake, and thus captures only one dimension of dietary exposure. In addition, sources of dietary sodium may differ across settings: in the United States, processed foods and foods consumed away from home often contribute substantially, whereas in China, home cooking and condiments (e.g., soy sauce) may play a more prominent role (32, 33). Accordingly, the amount of “total sodium exposure” represented by table salting may not be directly comparable across these dietary contexts (34). Therefore, when comparing the U.S. NHANES cohort with the Chinese clinical cohort, we focus on the consistency of association patterns (direction and reproducibility) rather than interpreting the findings as comparable dose–response relationships for overall sodium exposure.

Existing literature supports a multifaceted relationship between sodium intake and sleep. From a hemodynamic perspective, elevated sodium levels disrupt water-electrolyte homeostasis and activate the renin-angiotensin-aldosterone system (35), which may fragment sleep by increasing nocturnal urination frequency (36–38) or exacerbating fluid-retention-related obstructive sleep apnea (39, 40). Additionally, high sodium intake may impair cognitive functions essential for sleep stability (41). However, previous research has predominantly focused on these direct physiological effects. In contrast, our study highlights potentially relevant behavioral and psychological factors. By treating table salt use as a behavioral indicator, we hypothesize that salt-related dietary exposure may be linked to sleep not only through hemodynamic pathways but also through mental health status.

Our mediation analysis suggests that depressive symptoms may statistically account for part of the association between discretionary salt use and sleep outcomes. While high salt consumption exerts a direct influence on sleep, a significant portion of this risk is channeled through the exacerbation of depressive symptoms. Notably, the larger proportion of the total association statistically accounted for by depressive symptoms in the clinical cohort (55.8%) than in the general population suggests that the overlap between depressive symptoms and sleep disturbances may be more prominent in high-risk clinical settings. These mediation results should be interpreted cautiously because, in cross-sectional data, they represent a statistical decomposition rather than causal mediation. Residual confounding and reverse or bidirectional relationships among sleep, depressive symptoms, and discretionary salt use remain possible; therefore, the findings are exploratory and require confirmation in prospective studies with repeated measurements. Biologically, high sodium exposure has been hypothesized to influence neurochemical and neuroendocrine pathways involved in mood regulation and sleep architecture (30). Specifically, a high-salt preference is closely linked to the dopaminergic reward-processing system. Excessive salt consumption has been shown to alter dopamine synthesis and enhance the activity of dopaminergic neurons, which are central to both reward seeking and the regulation of circadian rhythms (42). From a neuropsychiatric perspective, dysregulation in these dopamine circuits is a shared pathological foundation for both depressive symptoms and sleep disturbances (43). Taken together, the observed triadic association among discretionary salt use, depressive symptoms, and sleep outcomes may be consistent with an underlying reward-related behavioral tendency, whereby frequent salt adding reflects not only physiological correlates of dietary habits but also a behavioral phenotype that could be associated with greater susceptibility to mood and sleep disturbances. Additionally, excessive sodium consumption is associated with disruption of the gut microbiota, which may trigger the production of inflammatory cytokines and induce neuroglial cell changes across the blood–brain barrier (44–48). These physiological stressors can trigger or worsen depressive symptoms, which in turn dysregulate the HPA axis and cortisol rhythms, ultimately leading to sleep disturbances. Therefore, depressive symptoms may not only co-occur with poor diet and sleep but may also be associated with the link between higher discretionary salt use and poorer sleep outcomes.

Subgroup analyses revealed that the association between salt use and sleep patterns is modified by demographic and lifestyle factors, with notable consistency across cohorts regarding age. The association was robustly observed in younger adults aged 20–45 years in both cohorts. Physiologically, younger individuals typically possess a more sensitive adrenergic system, allowing for rapid activation in response to sodium load, which directly impacts sleep structure (49, 50). In contrast, older adults often present with multiple comorbidities and polypharmacy, which may mask the independent effects of sodium intake. Regarding education, participants with a high school or equivalent education appeared most vulnerable. This may reflect a “middle-education trap”: these individuals lack the protective health literacy of the highly educated, yet unlike lower-education groups—whose complex and variable living conditions often overshadow specific dietary risks—their lifestyle patterns are stable enough for the adverse effects of salt to be statistically observable (51, 52).

Interestingly, lifestyle interactions displayed divergent patterns between the two cohorts regarding alcohol consumption. In the general population (NHANES), more frequent table salt use was significantly associated with unhealthy sleep among drinkers. Given that unhealthy sleep was less prevalent among drinkers in this cohort (Table 1), this pattern may reflect the “sick quitter” effect in the general population (where individuals with poor health abstain from alcohol), leaving a relatively healthy drinking group where the specific synergistic harm of alcohol and salt on neuroendocrine regulation becomes statistically prominent (53, 54). Conversely, in the external clinical replication cohort (Gansu Provincial People’s Hospital), the risk was concentrated among non-drinkers. This discrepancy supports the “self-medication” hypothesis prevalent in clinical settings: patients with severe symptoms (and thus poorer sleep) were significantly more likely to consume alcohol (Table 1), potentially to cope with their distress. This high baseline risk may reflect cohort-specific context and residual confounding, which could attenuate the observed association among drinkers; in contrast, the association may appear more evident among non-drinkers. In the general population, the observed association appeared stronger among alcohol users, suggesting that discretionary salt use and alcohol consumption may jointly mark higher-risk lifestyle profiles. In the psychiatric outpatient sample, the association was more evident among non-drinkers, indicating potential heterogeneity by drinking status in high-risk clinical settings. Similarly, stronger associations in non-hypertensive individuals may reflect differences in baseline risk profiles or treatment-related factors (e.g., antihypertensive use), although these subgroup findings should be interpreted cautiously given the observational design and possible residual confounding.

In two independent cohorts, more frequent discretionary table salt use was associated with an unhealthy sleep pattern, and depressive symptoms statistically accounted for part of this association. These findings are observational and hypothesis-generating rather than causal, and they motivate prospective longitudinal and intervention studies to test whether reducing discretionary salt use is followed by changes in depressive symptoms and sleep outcomes. Improved measurement of total sodium intake and objective sleep assessments will be important before translating these associations into clinical or public health recommendations.

This study has several strengths. We leveraged a dual-cohort design by combining a nationally representative U.S. sample (NHANES) with an independent external clinical replication cohort from Gansu Provincial People’s Hospital, providing evidence of reproducibility in a distinct clinical context. We further applied mediation analyses to quantify the statistical indirect component through depressive symptoms, offering hypothesis-generating insights. Methodological rigor was enhanced by the use of NHANES sampling weights and comprehensive adjustment for potential confounders in both cohorts.

However, this study has several limitations. First, the cross-sectional design precludes establishing temporality among discretionary salt use, depressive symptoms, and sleep outcomes; thus, the mediation findings should be interpreted as a statistical decomposition of associations rather than causal mediation. The mediation analyses should be considered exploratory because key assumptions (e.g., no unmeasured mediator–outcome confounding) cannot be verified in cross-sectional data. Second, table-salting frequency captures discretionary salt-use behavior but does not quantify total dietary sodium from cooking, processed foods, or condiments, which may introduce exposure misclassification and attenuate associations; moreover, differences in sodium sources and eating practices between the U.S. NHANES cohort and the Chinese clinical cohort may affect cross-cohort comparability. The external clinical replication cohort was convenience-sampled from psychiatric settings, so selection bias and inflated effect sizes are possible; thus, it supports reproducibility in a high-risk clinical context rather than population-level generalizability. Third, residual confounding cannot be excluded despite extensive adjustment. In particular, sleep- and occupation-related factors that were not consistently available or harmonizable across the NHANES cycles and the external clinical replication cohort (e.g., obstructive sleep apnea, shift work, and physical activity) may have contributed to residual confounding. Finally, sleep and dietary measures were self-reported and may be subject to recall bias. Future studies using prospective designs and standardized or objective sleep assessments are needed to clarify temporal ordering and inform evidence-based recommendations.

## 5 Conclusion

In conclusion, across a nationally representative NHANES sample and an independent external clinical replication cohort, more frequent discretionary table salt use was associated with higher odds of unhealthy sleep patterns, with depressive symptoms accounting for a substantial proportion of the total association. Importantly, table salt frequency reflects a behavioral marker of discretionary salt use and does not capture total dietary sodium intake from cooking or processed foods. Given the cross-sectional design, these findings should be interpreted as correlational and hypothesis-generating rather than evidence that reducing salt use will improve sleep. Prospective studies with repeated measures of sodium exposure and sleep, and randomized intervention trials targeting discretionary salt-use behavior, are needed to test temporal ordering and causal effects.

## Data Availability

The raw data supporting the conclusions of this article will be made available by the authors, without undue reservation.

## References

[1] Irwin MR. Sleep and inflammation: partners in sickness and in health. Nat Rev Immunol. (2019) 19:702–15. doi: 10.1038/s41577-019-0190-z, 31289370

[2] Benjafield AV Ayas NT Eastwood PR Heinzer R Ip MSM Morrell MJ . Estimation of the global prevalence and burden of obstructive sleep apnoea: a literature-based analysis. Lancet Respir Med. (2019) 7:687–98. doi: 10.1016/S2213-2600(19)30198-5, 31300334 PMC7007763

[3] Hirshkowitz M Whiton K Albert SM Alessi C Bruni O DonCarlos L . National Sleep Foundation’s sleep time duration recommendations: methodology and results summary. Sleep Health. (2015) 1:40–3. doi: 10.1016/j.sleh.2014.12.010, 29073412

[4] Ford ES Cunningham TJ Croft JB. Trends in self-reported sleep duration among US adults from 1985 to 2012. Sleep. (2015) 38:829–32. doi: 10.5665/sleep.4684, 25669182 PMC4402659

[5] Joshi K Cambron-Mellott MJ Costantino H Pfau A Jha MK. The real-world burden of adults with major depressive disorder with moderate or severe insomnia symptoms in the United States. J Affect Disord. (2023) 323:698–706. doi: 10.1016/j.jad.2022.12.005, 36481229

[6] Behrens A Anderberg P Berglund JS. Sleep disturbance predicts worse cognitive performance in subsequent years: a longitudinal population-based cohort study. Arch Gerontol Geriatr. (2023) 106:104899. doi: 10.1016/j.archger.2022.104899, 36512858

[7] Huyett P Siegel N Bhattacharyya N. Prevalence of sleep disorders and association with mortality: results from the NHANES 2009–2010. Laryngoscope. (2021) 131:686–9. doi: 10.1002/lary.28900 32681735

[8] Brandt J Leong C. Benzodiazepines and Z-drugs: an updated review of major adverse outcomes reported on in epidemiologic research. Drugs R D. (2017) 17:493–507. doi: 10.1007/s40268-017-0207-7, 28865038 PMC5694420

[9] Rémi J Pollmächer T Spiegelhalder K Trenkwalder C Young P. Sleep-related disorders in neurology and psychiatry. Dtsch Arztebl Int. (2019) 116:681–8. doi: 10.3238/arztebl.2019.0681, 31709972 PMC6865193

[10] Zuraikat FM Wood RA Barragán R St-Onge MP. Sleep and diet: mounting evidence of a cyclical relationship. Annu Rev Nutr. (2021) 41:309–32. doi: 10.1146/annurev-nutr-120420-021719, 34348025 PMC8511346

[11] Ren R Huang R Li Y Wang W Ye X Xi L . Depressive symptoms mediate the association between dietary inflammatory index and sleep: a cross-sectional study of NHANES 2005–2014. J Affect Disord. (2025) 372:117–25. doi: 10.1016/j.jad.2024.12.020, 39638055

[12] Godos J Grosso G Castellano S Galvano F Caraci F Ferri R. Association between diet and sleep quality: a systematic review. Sleep Med Rev. (2021) 57:101430. doi: 10.1016/j.smrv.2021.101430, 33549913

[13] St-Onge M-P Mikic A Pietrolungo CE. Effects of diet on sleep quality. Adv Nutr. (2016) 7:938–49. doi: 10.3945/an.116.012336, 27633109 PMC5015038

[14] Nedeltcheva AV Kilkus JM Imperial J Kasza K Schoeller DA Penev PD. Sleep curtailment is accompanied by increased intake of calories from snacks. Am J Clin Nutr. (2009) 89:126–33. doi: 10.3945/ajcn.2008.26574, 19056602 PMC2615460

[15] He FJ MacGregor GA. A comprehensive review on salt and health and current experience of worldwide salt reduction programmes. J Hum Hypertens. (2009) 23:363–84. doi: 10.1038/jhh.2008.144, 19110538

[16] Frank S Gonzalez K Lee-Ang L Young MC Tamez M Mattei J. Diet and sleep physiology: public health and clinical implications. Front Neurol. (2017) 8:393. doi: 10.3389/fneur.2017.00393, 28848491 PMC5554513

[17] Wilck N Matus MG Kearney SM Olesen SW Forslund K Bartolomaeus H . Salt-responsive gut commensal modulates T(H)17 axis and disease. Nature. (2017) 551:585–9. doi: 10.1038/nature24628, 29143823 PMC6070150

[18] Xu Y Wang W Wang M Liu X Lee MH Wang M . High salt intake attenuates breast cancer metastasis to lung. J Agric Food Chem. (2018) 66:3386–92. doi: 10.1021/acs.jafc.7b05923, 29553743

[19] Faraco G Brea D Garcia-Bonilla L Wang G Racchumi G Chang H . Dietary salt promotes neurovascular and cognitive dysfunction through a gut-initiated TH17 response. Nat Neurosci. (2018) 21:240–9. doi: 10.1038/s41593-017-0059-z, 29335605 PMC6207376

[20] Grandner MA Jackson N Gerstner JR Knutson KL. Sleep symptoms associated with intake of specific dietary nutrients. J Sleep Res. (2014) 23:22–34. doi: 10.1111/jsr.12084, 23992533 PMC3866235

[21] Murphy KR Deshpande SA Yurgel ME Quinn JP Weissbach JL Keene AC . Postprandial sleep mechanics in *Drosophila*. eLife. (2016) 5:e19334. doi: 10.7554/eLife.19334, 27873574 PMC5119887

[22] Verwey M Dhir S Amir S. Circadian influences on dopamine circuits of the brain: regulation of striatal rhythms of clock gene expression and implications for psychopathology and disease. F1000Res. (2016) 5:2062. doi: 10.12688/f1000research.9180.1, 27635233 PMC5007753

[23] Sandhu EC Fernando ABP Irvine EE Tossell K Kokkinou M Glegola J . Phasic stimulation of midbrain dopamine neuron activity reduces salt consumption. eNeuro. (2018) 5:ENEURO.0064-18.2018. doi: 10.1523/eneuro.0064-18.2018, 29766048 PMC5952649

[24] Xie J Wang D Ling S Yang G Yang Y Chen W. High-salt diet causes sleep fragmentation in young *Drosophila* through circadian rhythm and dopaminergic systems. Front Neurosci. (2019) 13:1271. doi: 10.3389/fnins.2019.01271, 31849585 PMC6895215

[25] Lopresti AL Hood SD Drummond PD. A review of lifestyle factors that contribute to important pathways associated with major depression: diet, sleep and exercise. J Affect Disord. (2013) 148:12–27. doi: 10.1016/j.jad.2013.01.014, 23415826

[26] McEwen BS Karatsoreos IN. Sleep deprivation and circadian disruption: stress, Allostasis, and allostatic load. Sleep Med Clin. (2015) 10:1–10. doi: 10.1016/j.jsmc.2014.11.007, 26055668 PMC8935364

[27] Kroenke K Spitzer RL Williams JB. The PHQ-9: validity of a brief depression severity measure. J Gen Intern Med. (2001) 16:606–13. doi: 10.1046/j.1525-1497.2001.016009606.x, 11556941 PMC1495268

[28] Kupfer DJ Kuhl EA Wulsin L. Psychiatry’s integration with medicine: the role of DSM-5. Annu Rev Med. (2013) 64:385–92. doi: 10.1146/annurev-med-050911-161945 23327527

[29] Zhou G Gan L Zhao B Fang F Liu H Chen X . Adding salt to foods and risk of psoriasis: a prospective cohort study. J Autoimmun. (2024) 147:103259. doi: 10.1016/j.jaut.2024.103259, 38823158

[30] Wang W Chang X Lin F Feng L Wang M Huang J . Adding salt to foods and risk of incident depression and anxiety. BMC Med. (2025) 23:32. doi: 10.1186/s12916-025-03865-x, 39838382 PMC11752635

[31] Li J Shang B Liu H Lin X Ning L Li F . Frequency of adding salt to food and risk of depression and anxiety: exploring the potential role of accelerated biological aging. J Affect Disord. (2025) 380:725–33. doi: 10.1016/j.jad.2025.03.179, 40174783

[32] Ahmed M Ng AP Christoforou A Mulligan C L’Abbé MR. Top sodium food sources in the American diet-using National Health and nutrition examination survey. Nutrients. (2023) 15:831. doi: 10.3390/nu15040831, 36839189 PMC9962803

[33] Du W Wang H Zhang J Zhang X Wei N Li Y . Sodium content of restaurant dishes in China: a cross-sectional survey. Nutr J. (2022) 21:10. doi: 10.1186/s12937-022-00762-4, 35177072 PMC8851779

[34] Anderson CAM Appel LJ Okuda N Brown IJ Chan Q Zhao L . Dietary sources of sodium in China, Japan, the United Kingdom, and the United States, women and men aged 40 to 59 years: the INTERMAP study. J Am Diet Assoc. (2010) 110:736–45. doi: 10.1016/j.jada.2010.02.007, 20430135 PMC4308093

[35] Verbalis JG. Renal physiology of nocturia. Neurourol Urodyn. (2014) 33:S6–9. doi: 10.1002/nau.22594, 24729151

[36] Ancoli-Israel S Bliwise DL Nørgaard JP. The effect of nocturia on sleep. Sleep Med Rev. (2011) 15:91–7. doi: 10.1016/j.smrv.2010.03.002, 20965130 PMC3137590

[37] Matsuo T Miyata Y Sakai H. Daily salt intake is an independent risk factor for pollakiuria and nocturia. Int J Urol. (2017) 24:384–9. doi: 10.1111/iju.13321, 28295650

[38] Vitiello MV Prinz PN Halter JB. Sodium-restricted diet increases nighttime plasma norepinephrine and impairs sleep patterns in man. J Clin Endocrinol Metab. (1983) 56:553–6. doi: 10.1210/jcem-56-3-553, 6822653

[39] Pimenta E Stowasser M Gordon RD Harding SM Batlouni M Zhang B . Increased dietary sodium is related to severity of obstructive sleep apnea in patients with resistant hypertension and hyperaldosteronism. Chest. (2013) 143:978–83. doi: 10.1378/chest.12-0802, 23288434 PMC3616687

[40] Fiori CZ Martinez D Gonçalves SC Montanari CC Fuchs FD. Effect of diuretics and sodium-restricted diet on sleep apnea severity: study protocol for a randomized controlled trial. Trials. (2015) 16:188. doi: 10.1186/s13063-015-0699-9, 25906818 PMC4411798

[41] Gildner TE Liebert MA Kowal P Chatterji S Snodgrass JJ. Associations between sleep duration, sleep quality, and cognitive test performance among older adults from six middle income countries: results from the Study on Global Ageing and Adult Health (SAGE). J Clin Sleep Med. (2014) 10:613–21. doi: 10.5664/jcsm.3782, 24932140 PMC4031401

[42] Hunter RW Dhaun N Bailey MA. The impact of excessive salt intake on human health. Nat Rev Nephrol. (2022) 18:321–35. doi: 10.1038/s41581-021-00533-0 35058650

[43] Chang Y Liu L Xu X Zhang S. The pathogenesis and treatment of insomnia combined with depression. Int J Gen Med. (2025) 18:4635–45. doi: 10.2147/ijgm.S547865, 40874239 PMC12379958

[44] Fan L Peng Y Wang J Ma P Zhao L Li X. Total glycosides from stems of *Cistanche tubulosa* alleviate depression-like behaviors: bidirectional interaction of the phytochemicals and gut microbiota. Phytomedicine. (2021) 83:153471. doi: 10.1016/j.phymed.2021.153471, 33636477

[45] Sampson TR Debelius JW Thron T Janssen S Shastri GG Ilhan ZE . Gut microbiota regulate motor deficits and neuroinflammation in a model of Parkinson’s disease. Cell. (2016) 167:1469–1480.e12. doi: 10.1016/j.cell.2016.11.018, 27912057 PMC5718049

[46] Zhang JC Yao W Dong C Yang C Ren Q Ma M . Blockade of interleukin-6 receptor in the periphery promotes rapid and sustained antidepressant actions: a possible role of gut-microbiota-brain axis. Transl Psychiatry. (2017) 7:e1138. doi: 10.1038/tp.2017.112, 28556833 PMC5534942

[47] Pan Y Chen XY Zhang QY Kong LD. Microglial NLRP3 inflammasome activation mediates IL-1β-related inflammation in prefrontal cortex of depressive rats. Brain Behav Immun. (2014) 41:90–100. doi: 10.1016/j.bbi.2014.04.007, 24859041

[48] Wang YL Han QQ Gong WQ Pan DH Wang LZ Hu W . Microglial activation mediates chronic mild stress-induced depressive- and anxiety-like behavior in adult rats. J Neuroinflammation. (2018) 15:21. doi: 10.1186/s12974-018-1054-3, 29343269 PMC5773028

[49] Ribera-Casado JM. Ageing and the cardiovascular system. Z Gerontol Geriatr. (1999) 32:412–9. doi: 10.1007/s003910050138, 10654379

[50] Strazzullo P D’Elia L Kandala NB Cappuccio FP. Salt intake, stroke, and cardiovascular disease: meta-analysis of prospective studies. BMJ. (2009) 339:b4567. doi: 10.1136/bmj.b4567, 19934192 PMC2782060

[51] Gallo LC Smith TW Cox CM. Socioeconomic status, psychosocial processes, and perceived health: an interpersonal perspective. Ann Behav Med. (2006) 31:109–19. doi: 10.1207/s15324796abm3102_2, 16542125

[52] Cutler DM Lleras-Muney A. Understanding differences in health behaviors by education. J Health Econ. (2010) 29:1–28. doi: 10.1016/j.jhealeco.2009.10.003, 19963292 PMC2824018

[53] Zheng D Yuan X Ma C Liu Y VanEvery H Sun Y . Alcohol consumption and sleep quality: a community-based study. Public Health Nutr. (2021) 24:4851–8. doi: 10.1017/s1368980020004553, 33183388 PMC11077453

[54] Ebrahim IO Shapiro CM Williams AJ Fenwick PB. Alcohol and sleep I: effects on normal sleep. Alcohol Clin Exp Res. (2013) 37:539–49. doi: 10.1111/acer.12006, 23347102

