# Supplementary material for: Association of table salt use with sleep patterns and depressive symptoms: population-based analysis with external clinical replication
Source: Front Nutr. 2026 Mar 25;13:1773531. doi: 10.3389/fnut.2026.1773531 (PMC13056815; doi:10.3389/fnut.2026.1773531)
Supplement: Supplementary file 1 [file Table_1.docx]

**Supplementary table 1.** Logistic regression between table salt use and Sleep disorder

|  | NHANES | | | Gansu Provincial People’s Hospital | | |
| --- | --- | --- | --- | --- | --- | --- |
| Table-salt use | Model 1 | Model 2 | Model 3 | Model 1 | Model 2 | Model 3 |
|  | OR (95% CI) *P*-value | OR (95% CI) *P*-value | OR (95% CI) *P*-value | OR (95% CI) *P*-value | OR (95% CI) *P*-value | OR (95% CI) *P*-value |
| Rarely/Occasionally | Reference | Reference | Reference | Reference | Reference | Reference |
| Often | 1.09 (0.90 ~ 1.32) 0.362 | 1.09 (0.90 ~ 1.32) 0.383 | 1.14 (0.94 ~ 1.39) 0.18 | 1.36 (0.81 ~ 2.28) 0.238 | 1.99 (1.08 ~ 3.68) 0.027 | 2.39 (1.21 ~ 4.73) 0.012 |

Model 1: unadjusted.

Model 2: Model 1 + age, gender, marital status
Model 3: Model 2 + smoking status, alcohol drinking status, CVD, diabetes, hypertension, and high cholesterol.

Abbreviations: CVD, cardiovascular disease.

**Supplementary table 2.** Logistic regression between table salt use and trouble sleeping.

|  | NHANES | | | Gansu Provincial People’s Hospital | | |
| --- | --- | --- | --- | --- | --- | --- |
| Table-salt use | Model 1 | Model 2 | Model 3 | Model 1 | Model 2 | Model 3 |
|  | OR (95% CI)  *P*-value | OR (95% CI)  *P*-value | OR (95% CI)  *P*-value | OR (95% CI)  *P*-value | OR (95% CI)  *P*-value | OR (95% CI)  *P*-value |
| Rarely/Occasionally | Reference | Reference | Reference | Reference | Reference | Reference |
| Often | 1.14 (1.01 ~ 1.28) 0.030 | 1.18 (1.05 ~ 1.33) 0.007 | 1.23 (1.09 ~ 1.39) <.001 | 1.39 (0.95 ~ 2.04) 0.009 | 1.74 (1.15 ~ 2.63) 0.009 | 1.95 (1.25 ~ 3.04) 0.003 |

Model 1: unadjusted.

Model 2: Model 1 + age, gender, marital status
Model 3: Model 2 + smoking status, alcohol drinking status, CVD, diabetes, hypertension, and high cholesterol.

Abbreviations: CVD, cardiovascular disease.

**Supplementary Table 3.** Linear regression between table salt use and sleep duration.

|  | NHANES | | | Gansu Provincial People’s Hospital | | |
| --- | --- | --- | --- | --- | --- | --- |
| Table-salt use | Model 1 | Model 2 | Model 3 | Model 1 | Model 2 | Model 3 |
|  | β (95% CI) *P*-value | β (95% CI) *P*-value | β (95% CI) *P*-value | β (95% CI)  *P*-value | β (95% CI) *P*-value | β (95% CI) *P*-value |
| Rarely/Occasionally | Reference | Reference | Reference | Reference | Reference | Reference |
| Often | -0.10 (-0.17 ~ -0.03) 0.005 | -0.09 (-0.16 ~ -0.02) 0.008 | -0.09 (-0.16 ~ -0.02)  0.008 | 0.13 (-0.16 ~ 0.42) 0.385 | -0.11 (-0.30 ~ 0.08) 0.248 | -0.23 (-0.43 ~ -0.03)  0.023 |


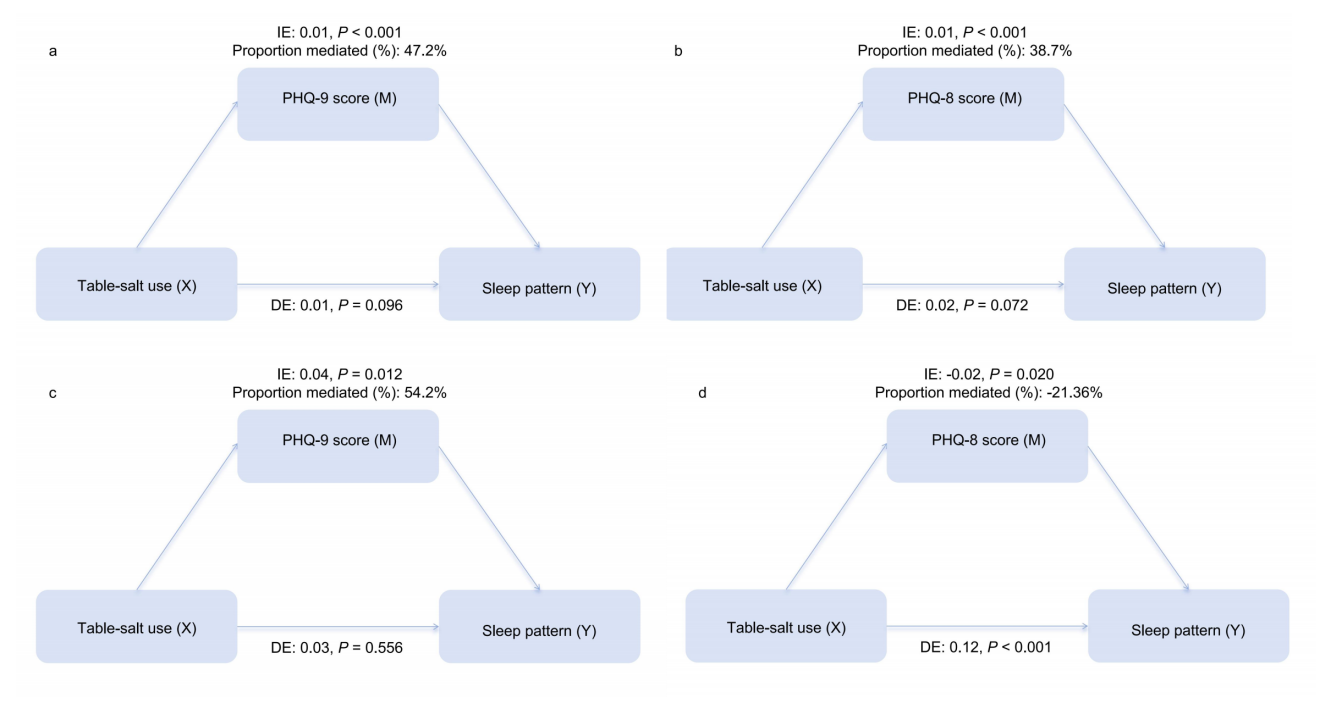


**Supplementary Figure 1.** Depressive symptoms were examined as a mediator to evaluate the mediation effect in the association between table-salt use (X) and sleep pattern (Y), with sensitivity analyses conducted to minimize measurement overlap. The upper panels show results from the NHANES cohort: (a) the mediator is the continuous PHQ-9 total score; (b) the mediator is the continuous modified PHQ-8 score, defined as the PHQ-9 total score excluding item 3 (the sleep item), to reduce overlap between depression measurement and sleep outcomes. The lower panels show results from the external clinical replication cohort: (c) the mediator is the continuous PHQ-9 total score; (d) the mediator is the continuous modified PHQ-8 score.


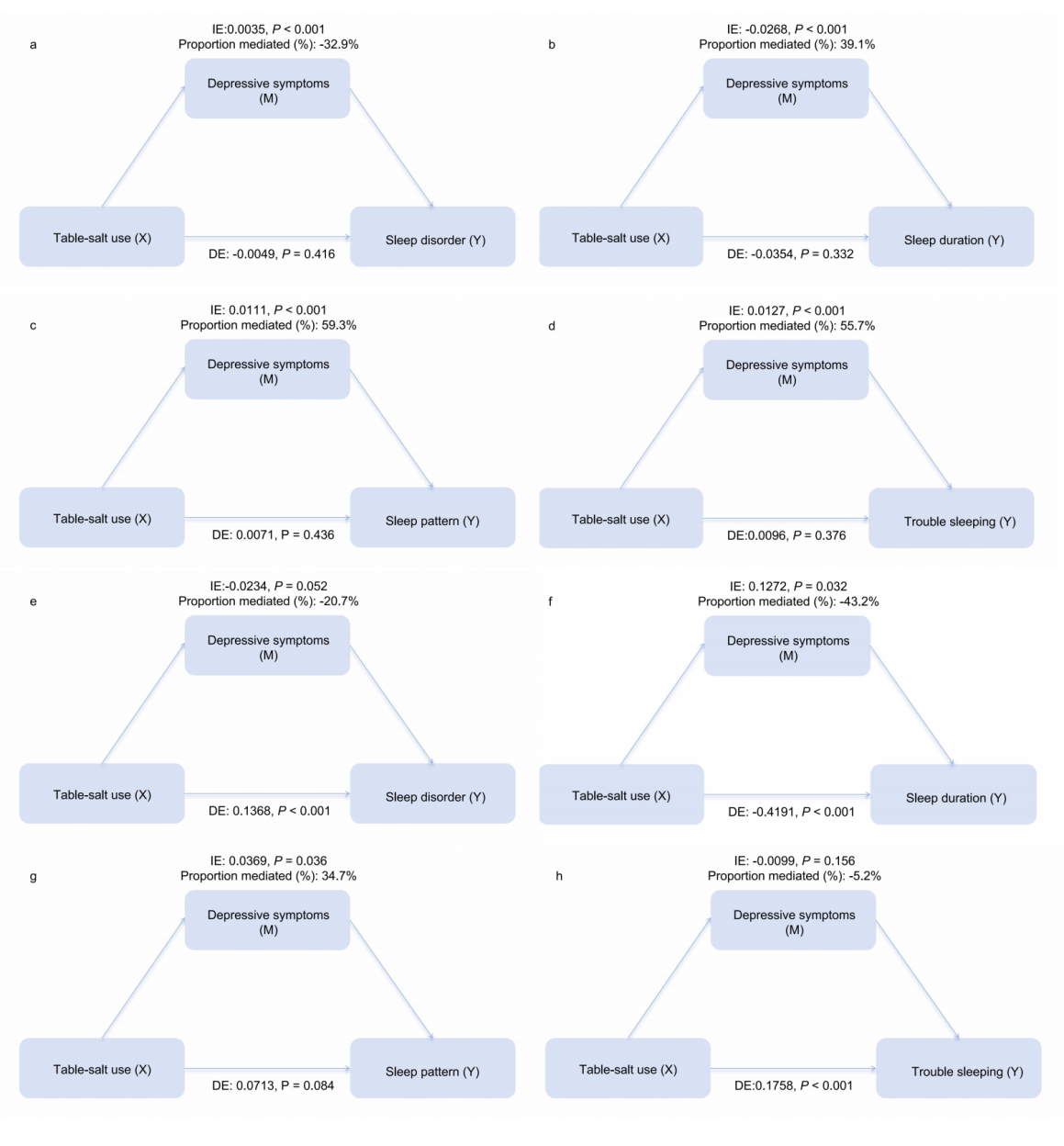


**Supplementary Figure 2.** Mediation analysis examining depressive symptoms as a mediator between the frequency of table salt use and sleep outcomes after excluding participants with baseline cardiovascular disease or diabetes. The upper panels display results from the NHANES cohort: (a) sleep disorder, (b) sleep duration, (c) sleep pattern, and (d) trouble sleeping. The lower panels display results from the Gansu Provincial People’s Hospital replication cohort: (e) sleep disorder, (f) sleep duration, (g) sleep pattern, and (h) trouble sleeping. Numbers on the paths represent the effect estimates. IE: Indirect Effect (pathway via depressive symptoms); DE: Direct Effect (pathway independent of depressive symptoms). Note: All mediation models were adjusted for age, gender, marital status, smoking status, alcohol drinking status, hypertension, and high cholesterol.
